# Thanks to all those who reviewed for *Journal of Medical Case Reports* in 2015

**DOI:** 10.1186/s13256-016-0842-6

**Published:** 2016-03-07

**Authors:** Michael R. Kidd

**Affiliations:** Faculty of Health Sciences, Flinders University, Adelaide, 5001, SA Australia

## Abstract

A peer-reviewed journal would not survive without the generous time and insightful comments of the reviewers, whose efforts often go unrecognized. Although final decisions are always editorial, they are greatly facilitated by the deeper technical knowledge, scientific insights, understanding of social consequences, and passion that reviewers bring to our deliberations. For these reasons, the Editor-in-Chief and staff of the *Journal of Medical Case Reports* warmly thank the 708 reviewers whose comments helped to shape the journal, for their invaluable assistance with review of manuscripts in Volume 9 (2015).

**Maria Ida Abbate**

Italy

**Ahmad Abdelkarim**

USA

**Mahmoud Abdelsalam**

USA

**Eman Abdelzaher**

Egypt

**Hassan Abduljabbar**

Saudi Arabia

**S Abrassart**

Switzerland

**Arun AC**

India

**Mulat Adefris**

Ethiopia

**Gauhar Afshan**

Pakistan

**Maryam Afshar**

USA

**Amitesh Agarwal**

USA

**Puneet Aggarwal**

India

**William Agger**

USA

**Varun Agrawal**

USA

**Abdul Razzaque Ahmed**

USA

**Fouad Al- Bayaty**

Malaysia

**Badr Al-Bawardy**

USA

**Bakr Alhussaini**

Saudi Arabia

**Abdullah Alhwiesh**

Saudi Arabia

**Yasser Al-Jehani**

Saudi Arabia

**Laith Alrubaiy**

UK

**Mohammad Al-Sabi**

Denmark

**Lisa Amir**

Australia

**Theodore Anagnostou**

Greece

**Olympia Anastasiou**

Germany

**Edwin J Anaya-Pava**

Mexico

**Mario Angelico**

Italy

**Alessandro Antonelli**

Italy

**Suresh Antony**

USA

**Stylianos Apostolidis**

Greece

**Alexandros Apostolopoulos**

Greece

**Edward Araujo Júnior**

Brazil

**Ripudaman Arora**

India

**Hammad Arshad**

USA

**Irene Asouhidou**

Greece

**Adolfo Attili**

Italy

**Awoniyi Awonuga**

USA

**Georgios Ayiomamitis**

Greece

**Irfan Ayub**

India

**Joseph Azuri**

Israel

**Dayanand Bagdure**

USA

**Anne Bakker**

Netherlands

**Nikolaos Baltayiannis**

Greece

**Bernardo Barahona-Correa**

Portugal

**Roberta Barbera**

Italy

**Kelli Barbour**

USA

**Alécio Barcelos**

Brazil

**Bilal Barkatali**

UK

**Giancarlo Barraco**

Italy

**Curtis Barry**

USA

**Özge Basaran**

Turkey

**Dan Bates**

Australia

**Himmatrao Bawaskar**

India

**Huseyin Bayazit**

Turkey

**Jasmina Begum**

India

**T Z Belhocine**

France

**Ahmed Belkouch**

Morocco

**Shannon Beres**

USA

**Peter Berlit**

Germany

**Christophe Berney**

Australia

**Alvise Berti**

Italy

**Rajesh Bhagat**

USA

**Rajesh Bhagat**

USA

**Sanjay Bhatia**

USA

**Dave Bhattacharya**

USA

**Anirban Bhattacharyya**

USA

**Debesh Bhoi**

India

**Zbigniew Binienda**

USA

**Rajshekhar Bipeta**

India

**Yochai Birnbaum**

USA

**Einar Bjornsson**

Iceland

**Miguel Blanca**

Spain

**Joerg Blessmann**

Germany

**Agnes Bloch-Zupan**

France

**Benoit Blondeau**

USA

**Benoit Blondeau**

USA

**Raphael Bodensohn**

Germany

**Jean-Jacques Boffa**

France

**Marianne Böni-Schnetzler**

Switzerland

**Kacy Bonnet**

USA

**Italo Borini**

Italy

**Christopher Boston**

USA

**Luca Botta**

Italy

**Stephen Boyle**

Australia

**Patrick Bradley**

UK

**Manjunatha BS**

India

**Sebina Bulic**

USA

**Klaus Burkhart**

Germany

**Larry Bush**

USA

**Mehmood Butt**

UK

**Cen Bytyqi**

Albania

**Carlo Caffarelli**

Italy

**Mario Camarda**

Italy

**Mehmet Akif Camkurt**

Turkey

**Giuseppina Campisi**

Italy

**David Cantu de Leon**

Mexico

**Joshua Carothers**

USA

**Jose Carreno**

Colombia

**Vincenzo Caruso**

UK

**Santos Castañeda**

Spain

**Roberto Casula**

UK

**Marco Cei**

Italy

**Bryan Chai**

USA

**Anthony W H Chan**

Hong Kong

**Yoon-Seok Chang**

Korea, South

**Woohyok Chang**

Korea, South

**Seho Chang**

Korea, South

**Arjun Chanmugam**

USA

**Ronnie Chee**

UK

**Jun Chen**

China

**M Chen**

China

**Y C Chiang**

Taiwan

**Toshimi Chiba**

Japan

**Joseph Choukroun**

France

**Stefano Cianetti**

Italy

**J Ciccolini**

France

**Grzegorz Cieslar**

Poland

**Frédéric Clarençon**

France

**William Clark**

Canada

**Yehuda Cohen**

USA

**Zachary Corbin**

USA

**Zachary Corbin**

USA

**Raul Corrales**

Chile

**Maria Lucia Correa-Giannella**

Brazil

**Anna Crocco**

Italy

**Sara Cruz**

Portugal

**Halisson Cruz**

Brazil

**Banushree CS**

India

**Kenji Cunnion**

USA

**M Cusidó**

Spain

**Hanady Daas**

USA

**Rongping Dai**

China

**Johanna Daily**

USA

**Giovanni Damiani**

Italy

**Adebiyi Damola**

UK

**Cem Dane**

Turkey

**Sambhunath Das**

India

**Krishanu Das**

India

**Fabrizio D'Ascenzo**

Italy

**Nandini Dave**

India

**Arka De**

India

**Chandima de Alwis**

Australia

**Guilherme de Jesus**

Brazil

**Marcelo Bezerra de Menezes**

Brazil

**Alexander Dechêne**

Germany

**Srihari Deepak**

UK

**Archie Defillo**

USA

**Hakkı Değer**

USA

**D Demellawy**

Canada

**Nihat Demir**

Turkey

**Rafael Denadai**

Brazil

**Omur Dereci**

Turkey

**Daniel Deschler**

USA

**Daniela Di Venere**

Italy

**Georgios Dimitrakakis**

UK

**Ioannis Dimitroulis**

Greece

**Charles Dinarelo**

USA

**Ramakant Dixit**

India

**Frank Domino**

USA

**Sarah Donaldson**

USA

**Marc Donath**

Switzerland

**Dimitrios Dougenis**

Greece

**Dimitrios Dragoumis**

UK

**Stamatoula Drakopoulou**

Greece

**Georgios Drosos**

Greece

**Ravi Durvasula**

USA

**Lee Eckhardt**

USA

**Hawa Edriss**

USA

**Ghada El Sagheer**

Saudi Arabia

**Ahmed El-Assmy**

Egypt

**Stephanie Elkins**

USA

**Adel El-Naggar**

USA

**Irene Esposito**

Germany

**Giuseppe Maria Ettorre**

Italy

**Mehtap Evran**

Turkey

**D Faas**

Germany

**Lee Fairlie**

South Africa

**Dimitrios Farmakiotis**

USA

**Francesca Farnetani**

Italy

**Hasan Fattah**

USA

**Stefano Fedele**

UK

**Steve Fenton**

USA

**Luis Fernández Salazar**

Spain

**Leslie Fiengo**

UK

**Anneliese Fischer Thom**

Brazil

**Joseph Fokam**

Cameroon

**Francois Fong**

Hong Kong

**T J Forbes**

USA

**Evangelia Fouka**

Greece

**Renato Franco**

Italy

**Laura Franco**

Italy

**Mary Fristad**

USA

**Ho Gwo Fuang**

Malaysia

**Clotilde Fuentes-Orozco**

Mexico

**Takaaki Fujii**

Japan

**Takashi Fujita**

Japan

**Takahiko Fukuchi**

Japan

**Koichi Fukunaga**

Japan

**Ioannis Galanis**

Greece

**Giovanni Galati**

Italy

**Danijel Galun**

USA

**Maurizia Gambacorta**

Italy

**Anthony Gannon**

USA

**Ann Gardner**

Sweden

**Bhavuk Garg**

India

**Rakesh Garg**

India

**Vishnu Garla**

India

**Roberto Gatto**

Italy

**Nurperi Gazioglu**

Turkey

**Marco Gazzoni**

Italy

**Ivan Gentile**

Italy

**Kemel Ahmed Ghotme Ghotme**

Colombia

**Stilianos Giannakopoulos**

Greece

**Kleanthis Giannoulis**

Greece

**Shah Giashuddin**

USA

**Rodney Gilbert**

UK

**Matteo Giorgi Pierfranceschi**

Italy

**Maria Gkika**

Greece

**Simona Glasberg**

Israel

**Jeroen Goede**

Switzerland

**Atul Goel**

India

**Elizabeth Golesic**

Canada

**Sripad Gopalakrishna Mehandale**

USA

**Ganesh Gopalakrishnan**

India

**David Gorard**

UK

**Karen Gordon**

UK

**Steven Goudy**

USA

**Lajya Goyal**

India

**Tarang Goyal**

India

**Barbara Gracious**

USA

**Josep M Grau**

Spain

**Andrew Green**

Ireland

**Adi Greenberg**

Israel

**Neil Greening**

UK

**Alistair Grey**

UK

**David Griffith**

USA

**Paraskevas Grivas**

Greece

**Gerald Gruber**

Austria

**Paolo Guarascio**

Italy

**W Gudu**

Ethiopia

**Nazmi Gultekin**

Turkey

**Deepak Gunjan**

India

**Gaurav Gupta**

USA

**Nitin Gupta**

India

**D K Gupta**

UK

**Anju Gupta**

India

**Vijay Hadda**

India

**Takahiro Haga**

Japan

**Christlieb Haller**

Switzerland

**Tsukasa Hanemoto**

Japan

**Guy Hans**

Belgium

**Noboru Hara**

Japan

**Kiyoshi Harada**

Japan

**Uma Hariharan**

India

**Leanne Harling**

UK

**Aoife Healy**

UK

**Tomás Henlín**

Czech Republic

**Marion Henry**

USA

**Manuel Hernandez-Guerra**

Spain

**Sarah Heron**

Australia

**Jesica Herrick**

USA

**Farhad Heydarian**

Iran

**Catherine Hill**

Australia

**Melissa Hilmes**

USA

**Konrad Hoetzenecker**

Austria

**Johannes Hofer**

Austria

**Shuji Horibe**

Japan

**Hironobu Hoshino**

Japan

**Dingming Huang**

China

**Caren Hughes**

USA

**Nexhmi Hyseni**

Kosovo

**Akihiko Ikeda**

Japan

**Munir Iqbal**

Canada

**Ahaorvi Isa**

Canada

**Mitsuaki Ishida**

Japan

**Hideya Ito**

Japan

**Ali Izadpanah**

Canada

**Hossain Jabbari**

Austria

**Janet Jacobson**

USA

**Yves Jacquemyn**

Belgium

**Sujit Jagtap**

India

**Vivek Jain**

UK

**Neetu Jain**

India

**Praveen Jajoria**

USA

**Bawo James**

Nigeria

**Vijayakumar Javalkar**

USA

**Hemangi Jerajani**

India

**Kathie-Ann Joseph**

USA

**Bharti Joshi**

India

**Milind Joshi**

India

**Matthias Jürgens**

Germany

**Stefanos Kachrilas**

United Arab Emirates

**Korhan Kahraman**

Turkey

**H Kaimakliotis**

USA

**Nikolaos Kalinderis**

Greece

**Kivanc Kamburoglu**

Turkey

**Kazuaki Kameda**

Japan

**Gokul Vignesh Kanda Swamy**

UK

**Takahiro Kanno**

Japan

**Paige Kaplan**

USA

**Neha Kapoor**

UK

**Levent Kaptanoglu**

Turkey

**Rie Karasawa**

Japan

**Manmeet Kaur**

USA

**Leticia Kawano-Dourado**

Brazil

**Joelle Kefer**

Belgium

**Jessica Keim-Malpass**

USA

**Martina Kelly**

Canada

**Y Keren**

USA

**Isaak Kesisoglou**

Greece

**Mohamed Kharfan-Dabaja**

USA

**Mahmoud Khattab**

Egypt

**Ayaz Khawaja**

USA

**Divya Khosla**

India

**Eva Kieslichová**

Czech Republic

**Wook Kim**

Korea, South

**Kumiko Kimura**

Japan

**Sandor Klis**

Netherlands

**Celebi Kocaoglu**

Turkey

**Christian Koch**

USA

**Maurizio Koch**

Italy

**Daniel Kokong**

Nigeria

**M K Kolar**

UK

**Likurgos Kolilekas**

Greece

**Eija Könönen**

Finland

**Z Korzets**

Israel

**Jan Kotarski**

Poland

**Kalliopi Kotsa**

Greece

**Vasileios Kouranos**

Greece

**Ioannis Koutelidakis**

Greece

**Gopal Kowdley**

USA

**Tomohide Koyama**

Japan

**Ali Koyuncuer**

Turkey

**Somasekhar Krishna**

USA

**Karen Krogfelt**

Denmark

**Bindu Kulshreshtha**

India

**Priya Kumthekar**

USA

**Jujju Kurian**

India

**Ali Kurt**

USA

**Andreas Kurtz**

Germany

**Kathryn Kvam**

USA

**Bernice Kwong**

USA

**Dimitrios Kyparissopoulos**

UK

**Stephen E Lapinsky**

Canada

**José Luis Lázaro Martínez**

Spain

**Milan Lepic**

Serbia

**Aaron Lesher**

USA

**Claudio Letizia**

Italy

**Roland Leung**

Hong Kong

**Steven Lewis**

USA

**Philippe Lheureux**

Belgium

**Bo Li**

Canada

**Zhiming Li**

China

**Gordon Li**

USA

**Chien-Yu Lin**

Taiwan

**Kuan-Chou Lin**

Taiwan

**Katerina Linhartova**

Czech Republic

**Michael Linnebank**

Switzerland

**Jules Lipoff**

USA

**Jing Liu**

USA

**Steven Livesey**

UK

**Lorenzo Lo Muzio**

Italy

**Martín Lorenzo**

Spain

**Marta Losa Iglesias**

Spain

**Peter Lu**

Singapore

**Chin-Fang Lu**

Taiwan

**Gilberto Luna Lugo**

Mexico

**Jicheng LV**

China

**Sally Ann Lynch**

Ireland

**Ian Mackenzie**

UK

**Karan Madan**

India

**Brett Mador**

Canada

**Marc Maegele**

Germany

**Gaetano Magro**

Italy

**Anand Mahadevan**

USA

**Ashoka Mahapatra**

India

**Mohamad Mahmoud**

USA

**Michael Mahmoudi**

UK

**Matthias Maiwald**

Singapore

**Chloe Mak**

Hong Kong

**Vijay Malpathak**

India

**Effrosyni Manali**

Greece

**Ayse Tulin Mansur**

Turkey

**Nizar Maraqa**

USA

**Dan Marek**

Czech Republic

**Athanasios Marinis**

Greece

**Konstantinos Markou**

Greece

**Henri Marret**

France

**Ian Marshall**

Australia

**Thomas Marth**

Germany

**Haruhiko Maruyama**

Japan

**Helena Marzo-Ortega**

UK

**Gwinyai Masukume**

Zimbabwe

**Joseph Kunju Mathew**

India

**Ranjana Mathur**

Singapore

**Shinya Matsuzaki**

Japan

**Valeria Mazzi**

Italy

**Federico Mecacci**

Italy

**Bruno Megarbane**

France

**Markus Meissner**

Germany

**Renuka Melkundi**

India

**Moises Mercado**

Mexico

**Elif Meseci**

Turkey

**Ahmed Messallam**

USA

**Linda Metaxa**

Italy

**Pavel Michalek**

Czech Republic

**Antonios Michalopoulos**

Greece

**Nickolaos Michalopoulos**

Greece

**George Michalopoulos**

Greece

**Ioannis Mikoniatis**

Greece

**Jeronimo Milano**

Brazil

**Kostas Mimidis**

Greece

**Bushra Mina**

USA

**Alireza Minagar**

USA

**Ravinder Minhas**

India

**Tammy Mirensky**

USA

**Hidekazu Misaki**

Japan

**Georgios-Alexandros Mistriotis**

UK

**Jun Miyauchi**

Japan

**Consuelo Modesto**

Spain

**Kathryn Moffett**

USA

**Mohamed Ali Ahmed Mohamed**

Egypt

**Amr Mohamed**

UK

**Shahida Mohd-Said**

Malaysia

**George Moll**

USA

**Guillermo Monsalve**

Colombia

**Tom Moreels**

Belgium

**Akira Morimoto**

Japan

**Naoto Morimura**

Japan

**Michael Moritz**

USA

**Karwan Moutasim**

UK

**Theodoros Moysidis**

Germany

**Nicola Mumoli**

Italy

**Zeynel Mungan**

Turkey

**William Murk**

USA

**Michelle Murray**

Ireland

**Natalia Myakova**

Russian Federation

**Eleni-Aikaterini Nagorni**

Greece

**Satoshi Nagoya**

Japan

**Manu Nair**

UK

**K Naito**

Japan

**R Nambron**

USA

**Tsutomu Namikawa**

Japan

**Abdulrasheed Nasir**

Nigeria

**Ramon Navarro**

United Arab Emirates

**Rodrigo Navarro-Ramirez**

Mexico

**Umit Nayki**

Turkey

**Ujjwal Neogi**

Sweden

**Nnabuike Chibuoke Ngene**

South Africa

**Peggy Nguyen**

USA

**Yuzuru Niibe**

Japan

**Hiroaki Nishioka**

Japan

**Xiaohui Niu**

China

**Joseph NM**

India

**Francisco Nogales**

Spain

**Maria Claudia Nogueira Zerbini**

Brazil

**Takehiro Noji**

Japan

**Kandai Nozu**

Japan

**Dimitrios Ntelios**

Greece

**Achilleas Ntinas**

Greece

**Anthony Nyamache**

Kenya

**F Ochsendorf**

Germany

**Onyema Ogbuagu**

USA

**Aung Oo**

UK

**Norberto Ortego-Centeno**

Spain

**Eleonora Ortu**

Italy

**Zdenko Ostojic**

Bosnia and Herzegovina

**Louise Pacher Hoffmann**

Brazil

**Vinesh Padayachy**

South Africa

**Sukhmani Padda**

USA

**Otávio Pagin**

Brazil

**Yatan Pal**

USA

**Ajay Pal Singh**

India

**Periklis Panagopoulos**

Greece

**Ravinder Pandey**

India

**Vasileios Papadopoulos**

Greece

**Kostas Papagiannopoulos**

UK

**Nikolaos Papanas**

Greece

**Theodossis S Papavramidis**

Greece

**Spyros Papiris**

Greece

**Yael Paran**

Israel

**Jin Kyun Park**

Korea, South

**Nigel John Parr**

UK

**Valentina Pastore**

Italy

**Athanasia Pataka**

Greece

**Jigisha Patel**

UK

**Konstantinos Paterakis**

Greece

**Sandip Pati**

USA

**Anil Patki**

India

**John Pauling**

UK

**S Paydas**

Turkey

**Deniz Peker**

USA

**Erdal Peker**

Turkey

**Brenton Pennicooke**

USA

**Judith Pentz**

USA

**Katy Peters**

USA

**Paul PG**

India

**Andrea Phillipou**

Australia

**Adriano Piattelli**

Italy

**Giorgina Barbara Piccoli**

Italy

**Michael Pitiakoudis**

Greece

**Constantinos Pitsios**

Greece

**Roy Poblete**

USA

**Andreas Polydorou**

Greece

**Mike Polyzonis**

Greece

**Anna Potulska-Chromik**

Poland

**Harry Powell**

UK

**Mukhyaprana Prabhu**

India

**Antonino Michele Previtera**

Italy

**Xingshun Qi**

China

**You-Wen Qian**

USA

**Waris Qidwai**

Pakistan

**Stuart Quan**

USA

**Keerthi Ramesh**

India

**Leonardo Ramirez Arreola**

Mexico

**Sahaj Rathi**

India

**Sahaj Rathi**

USA

**Amir Ravandi**

Canada

**Daniel Raymond**

USA

**P Reper**

Belgium

**Benjamin Rhodes**

UK

**Susan Richardson**

Canada

**Daniel Riche**

USA

**John Ringman**

USA

**Richard Rison**

USA

**Nicoletta Riva**

Italy

**Sandro Rizoli**

Canada

**Michele Roberts**

USA

**Paul Robinson**

UK

**Raffaele Rocchi**

Italy

**Manuel Rodriguez-Davalos**

USA

**Udo Rolle**

Germany

**Ilaria Romani**

Italy

**Damian Romero**

USA

**Lorraine Rosamilia**

USA

**Barry Rosenbloom**

USA

**Lionel Rostaing**

France

**Jessica Roybal**

USA

**Mourelatou Roza**

Greece

**Andres Mariano Rubiano Escobar**

Colombia

**Vidar Ruddox**

Norway

**Antonio E Ruiz Serrato**

Spain

**Jaime Ruiz -Tovar**

Spain

**Clark Russell**

UK

**Durre Sabih**

Pakistan

**Ahmed-Ramadan Sadek**

UK

**Ali Sadrizadeh**

Iran

**Arun Saini**

USA

**Atsushi Sakuraba**

USA

**Rosa Salazar**

Spain

**Nikolaos Salemis**

Greece

**Sundeep Saluja**

India

**Jayanta Samanta**

India

**Nicolas Sananes**

France

**Mario Santini**

Italy

**Vidyadhar Sardesai**

India

**Shinya Sato**

Japan

**Paola Savoia**

Italy

**Ilyas Sayar**

USA

**Francesco Sbrana**

Italy

**Bernhard Schaller**

UK

**Bernhard Schaller**

France

**Richard Scher**

USA

**Ellen Scherl**

USA

**Breno Schor**

Brazil

**Jan Schroeder**

Italy

**Cara Sedney**

USA

**Marta Seghieri**

Italy

**Virendra N Sehgal**

India

**Ali Seifi**

USA

**Francis Seow-Choen**

Singapore

**David Seres**

USA

**Arnold Seto**

USA

**Utkan Sevuk**

Turkey

**Daniel Alexander Shaerf**

UK

**Daniel Shaerf**

UK

**Lokesh Shahani**

USA

**Lee Shapiro**

USA

**Alka Sharma**

India

**Shilpa Sharma**

India

**Aparna Sharma**

India

**Shakti Sharma**

India

**Dominick Shaw**

UK

**Susan Shaw**

USA

**Manjunath Shenoy**

India

**Jiangang Shi**

China

**Nektaria Sidiropoulou**

Greece

**Matthew Siedhoff**

USA

**Navneet Singh**

India

**D A Sippo**

USA

**Panagiotis Skendros**

Greece

**Thomas Skripuletz**

Germany

**Samantha Sloan**

UK

**Stuart Smith**

UK

**Ioannis Sokolakis**

Greece

**Jean-Karl Soler**

Malta

**Cristina Solomon**

Austria

**Amey Sonavane**

India

**Gianluca Sottilotta**

Italy

**Vasileios Souftas**

Greece

**Karen Speck**

USA

**John Spurzem**

USA

**Thambipillai Sri Paran**

Ireland

**James Stefanou**

UK

**Paschalis Steiropoulos**

Greece

**Sara Stern**

USA

**Jose Subauste**

USA

**Anbukkani Subbian**

India

**Koichiro Sugimura**

Japan

**Tae-Yun Sung**

Korea, South

**Bhaskar Suryanarayanan**

India

**Akio Suzumura**

Japan

**Mathias Tiedemann Svendsen**

Denmark

**Yechiel Sweed**

Israel

**Clodagh Sweeney**

Ireland

**Brian Swick**

USA

**Federico Tacconi**

Italy

**Guillaume Taieb**

France

**Arturo Tamayo**

Canada

**Bien Keem Tan**

Singapore

**Yoshinari Tanaka**

Japan

**Giovanni Tarantino**

Italy

**Phil Taussky**

USA

**Marco Teli**

Italy

**Irene Terzi**

Greece

**Sidney Tessler**

USA

**Jagdeep Thakur**

India

**Loukas Thanos**

Greece

**Mark Thomas**

New Zealand

**Nicholas Todd**

UK

**Yukiharu Todo**

Japan

**Esteban Torche**

Chile

**Paschalis Tossios**

Greece

**Silvia Trifiro**

Italy

**Stavros Tryfon**

Greece

**Athina Tsili**

Greece

**Bernard Tuch**

Australia

**Mutahir Tunio**

Saudi Arabia

**Vasilios Tzilas**

Greece

**A Udelnow**

Germany

**M Iftekhar Ullah**

USA

**Anayat Ullah**

UK

**Nurcan Unver**

Turkey

**Vijai Upadhyaya**

India

**Shinji Urakami**

Japan

**Nicolás Useche**

Colombia

**Sinan Uzman**

Turkey

**Kamen Valchanov**

UK

**Alejandra Valenzuela**

USA

**Virginia van Duyne**

USA

**Robert-Jan van Geuns**

Netherlands

**Ivor Vanhegan**

UK

**Vittorio Vanini**

Italy

**John Vaughn**

USA

**Anand Venkatraman**

USA

**Giovanni Vennarecci**

Italy

**Abhai Verma**

India

**Serena Vettori**

Italy

**Kenneth Vick**

USA

**Nicolas Vignier**

France

**Kumar Vijay**

India

**Roberto Villani**

Italy

**Ana Paula Vivas**

Brazil

**Mavroudis Voultsos**

Greece

**Stergios Vradelis**

Greece

**Spyridon Vrakas**

Greece

**Zoran Vratnica**

Montenegro

**Sidhesh Wagh**

India

**Heather Wakelee**

USA

**Mohammad Sami Walid**

USA

**Elaine Walsh**

Ireland

**Stephen Walsh**

USA

**Zhongxiao Wang**

USA

**Imtiaz Wani**

India

**Orli Wargon**

Australia

**Toru Watanabe**

Japan

**Laura Waters**

UK

**Rafal Watrowski**

Germany

**Katrin Weier**

Canada

**Huang Wen-Bin**

China

**Sandra Werner**

USA

**Elspeth Whitby**

UK

**Jill Whitehouse**

USA

**Timothy Wiegand**

USA

**Michael Winkelman**

Brazil

**Grace Wong**

Hong Kong

**X Wu**

China

**Daisuke Yabe**

Japan

**Sachin Yallappa**

UK

**Satoru Yamada**

Japan

**Sohsuke Yamada**

Japan

**Yan Yan**

China

**Toru Yanagawa**

Japan

**Huilin Yang**

China

**Bienvenido Yangco**

USA

**Yukihiro Yano**

Japan

**Danielle Yanuck**

USA

**Mohamed Yassin**

USA

**Thomas Yau**

Hong Kong

**Edward Young**

USA

**Selcuk Uksel**

Turkey

**Sadik Yurttutan**

Turkey

**Zacharias Zachariah**

Switzerland

**Marco A Zanini**

Brazil

**Paul Zarogoulidis**

Greece

**Jianquan Zhang**

China

**Ling Zhang**

USA

**Dongsheng Zhou**

China

**Vas Zochios**

UK

**Abdolali Zolghadrasli**

Iran

**Alauldeen Zubair**

Iraq

